# How health system failures and prevailing social norms drive mistreatment of women in maternity care in Ethiopia: a qualitative study

**DOI:** 10.1080/16549716.2025.2526890

**Published:** 2025-07-08

**Authors:** Habtamu Kasaye, Vanessa Scarf, Annabel Sheehy, Kathleen Baird

**Affiliations:** a Collectives of Midwifery, Child and Family Health, School of Nursing and Midwifery, Faculty of Health, University of Technology Sydney, Sydney, New South Wales, Australia; b Midwifery Department, School of Nursing and Midwifery, Institute of Health Science, Wollega University, Nekemte, Ethiopia

**Keywords:** Mistreatment of women, qualitative study, health system failure, social norms, violence against women

## Abstract

**Background:**

Mistreatment of women during maternity care is a widespread global issue, particularly in low- and middle-income countries where health disparities intersect with rigid gender norms, systemic inequality and domestic violence.

**Objectives:**

This paper aims to explore how health system failures and societal norms against women contribute to the mistreatment of women during maternity care.

**Methods:**

A qualitative study was conducted among maternal healthcare providers in the East Wollega Zone, Ethiopia. In-depth interviews with purposively selected participants in Afan Oromo, each lasting 30–60 min, were conducted until data saturation was reached at 20 interviews. The interviews were audio-recorded, transcribed, translated into English, coded using NVivo 12 and analysed through thematic analysis guided by the Socioecological and Quality of Care frameworks.

**Results:**

Health system conditions and constraints, such as under-resourcing and issues related to governance and providers’ prejudices shaped by societal norms, were found to drive mistreatment of women during maternity care. These drivers manifest at various levels throughout the healthcare system, including personal, interpersonal, facility-level, health system and societal dimensions. Overcrowding, staff shortages and low pay led to burnout, which eventually resulted in mistreatment of women. The lack of recognition from administrators and professional hierarchies also added to provider frustration, which was sometimes directed at women. Societal issues, like gender-based violence, further influenced these behaviours.

**Conclusion:**

Mistreatment of women during maternity care continues to hinder the delivery of quality care. Addressing gender inequality, boosting healthcare worker motivation and ensuring fair treatment among staff are essential for promoting respectful maternity care.

## Paper context

**Main findings:** This study presents a qualitative study that involved maternal healthcare providers to explore the underlying drivers of the mistreatment of women during maternity care in western Ethiopia through integrating data obtained from participants with the socioecological frameworks for violence against women and WHO’s Quality of Care.

**Added knowledge:** The interpretive qualitative analysis identified factors contributing to the mistreatment of women stemming from individual, interpersonal, institutional, systematic and societal origins. These drivers are manifestations of broader issues in the under-resourcing, governance and social norms in the study area, which demand strategic actions to fully prevent the mistreatment of women in health facilities during maternity care.

**Global health impact for policy and action:** This study offers insights to inform health policies and practices to improve service delivery by fostering a health system that supports its caregivers, engages communities and eliminates gender-based violence.

## Background

Maternal and child health challenges compound the difficulties of healthcare provision in developing countries, alongside a growing burden of non-communicable diseases and the continuing struggle with infectious diseases [1]. Efforts to address these healthcare challenges must focus on the interplay of key underlying factors such as poverty, which contributes to infrastructural limitations and financial instability, an inadequate and under-capacitated workforce and disparities in the provision of essential health services [2]. The intersection of these factors often leads to persistent health inequalities in low- and middle-income countries (LMICs), particularly in maternal and child health, where maternal and child mortality rates are disproportionately high compared to the developed world [3]. While preventing mortality and morbidity should remain a priority, maternity care provision must also focus on delivering women-centred, holistic, high-quality care in an integrative manner to ensure positive experiences for all [4].

Mistreatment of women during maternity care is a significant factor that negatively affects women’s positive experiences during pregnancy and childbirth [5]. It has been recognised as a major global maternal health issue [5], with its prevalence ranging from one in every ten women in Australia [6] to two in five women in sub-Saharan Africa [7]. Such mistreatment can violate basic human and patient rights and lead to severe psychological and physical consequences for both mothers and newborns [8], such as postpartum depression [9,10] and birth injuries [11]. These negative outcomes are often worsened by power imbalance between healthcare providers (HCPs) and women through creating a hierarchy that undermines the autonomy of the women, conditioning them to trust in care givers’ authority and knowledge and normalisation of mistreatment [12]. Furthermore, in settings where health disparities intersect with socio-cultural issues such as rigid gender norms, gender inequality and intimate/domestic violence against women, the risk of mistreatment is even higher [13]. Additionally, factors like normalisation of various forms of violence in society, whether it is public violence, violence against children or even civil unrest, can influence how HCPs deliver their services [14,15]. It can create an environment of fear and insecurity, leading to stress and burnout, and further embed mistreatment within healthcare settings [14].

A range of other factors can contribute to the mistreatment of women and their families during maternity care, even though most HCPs typically offer empathetic care, understanding their patients’ pain and suffering, while maintaining emotional boundaries [16]. In LMICs, many health facilities and professionals work under immense pressure due to underfunding, limited resources and overwhelming demand for their services [1]. These challenging conditions can often blur the lines between HCPs and patients, resulting in indifference or inadequate responses from professionals to the needs and expectations of women and their families. Healthcare providers in Ethiopia face dual challenges: working in under-resourced settings and combating societal gender-based oppression against women. Operating in an under-resourced environment not only poses challenges in delivering services but also affects the resilience of HCPs, frequently leading to burnout and reduced quality of care as a result of compassion fatigue and leading them to behave in a disrespectful and abusive manner [17,18]. In addition to resource constraints, issues such as strained relationships among various HCPs, inadequate fundings, and a lack of recognition can further impact the quality of care offered [19].

In Ethiopia, violence against women is another macro-level issue that has been normalised at a societal level, with various forms of violence, physical, sexual, and emotional, affecting up to 4–34% of the women. This violence is often perpetrated by male partners or others, alongside non-consensual and unlawful child marriages [20]. Healthcare providers are also part of society, regardless of their gender; these harmful societal practices can influence their behaviour towards women in the healthcare facilities where they work. This is especially true when these societal norms intersect with challenges within the health system, such as resource shortages and poor governance by managers, which can all lead to the normalisation of mistreatment and violence.

The normalisation of violence against women, combined with systemic health inequalities, creates an environment where mistreatment during maternity care is more likely to occur and persist. Addressing these deeply rooted societal norms, along with improving the health system’s infrastructure and governance, is critical to reducing mistreatment and enhancing the overall quality of maternal healthcare. While understanding how societal gender inequality, resource limitations and professional dynamics all contribute to mistreatment is essential for informing policy and practice, evidence in this area remains limited. This qualitative study explored how health systems, governance, inter-professional relationships and social norms contribute to the mistreatment of women in health facilities, based on data from HCPs in Western Ethiopia.

## Methods

### Study design, context and population

In this study, we present qualitative findings from a larger mixed-methods study conducted on the mistreatment of women during maternity care in the East Wollega Zone, Western Ethiopia, from women’s and HCPs’ perspectives from February to December 2022. The qualitative data from HCPs, including midwives, nurses, physicians (Obstetrician and Gynaecologists) and health officers (also known as public health officers are non-physician clinicians in Ethiopia serving both clinical and public health services, mostly in primary healthcare units of rural areas) [21]. The HCPs who participated in the interviews have been working in hospitals and health centres with their working areas including antenatal care, labour and birth and post-natal units regardless of their gender identity, including both male and female professionals.

### Description of frameworks used

Guided by a pragmatist worldview, data from HCPs were analysed using two frameworks: the Socioecological Framework (SEF) for violence against women [22] and the World Health Organization’s (WHO) Quality of Care (QoC) framework for maternal and child health [23]. The SEF conceptualises gender-based violence as a multifaceted phenomenon influenced by personal, situational and socio-cultural factors [22]. Meanwhile, the WHO’s Quality of Care Framework [23], a conceptual framework that outlines how the quality of care provided to women and newborns can be improved using a structure–process–outcome model.

These two frameworks can elucidate the multilevel determinants and comprehensive dimensions of mistreatment within maternity care. WHO’s QoC framework identifies the importance of having competent and motivated human power and essential physical resources, as well as good leadership/governance in the health system, which acts as the building blocks to ensure quality of care [23]. The lack/inadequacy of these essential components leads to poor quality of care for women and newborns. The attitude and behavioural parts that drive the mistreatment of women, especially during critical periods of pregnancy and childbirth, are parallel to the violence towards women in society. The integration of both frameworks provided a theoretical foundation and structured lens for interpreting and understanding the mistreatment of women, particularly emphasising instances of mistreatment not only as a result of abusive actions by HCPs but also as the consequences of health system constraints, including governance/leadership in the health system and behaviours that could be influenced by violence in society.

In this study, mistreatment of women during maternity care in health facilities refers to various forms of interpersonal abuse (such as physical abuse, verbal abuse, stigma and discrimination), as well as other types of mistreatment, including failure to meet professional standards of care, poor rapport between women and HCPs and health system conditions and constraints, as outlined in the WHO research group’s systematic review [5].

### Recruitment and sampling

Following the completion of a self-administered survey [19], HCPs were invited to participate in in-depth interviews. The selection criteria targeted diverse professions within the maternity care sector, various experiences, facility location and levels of care, including health care centres, district hospitals and referral/specialised hospitals. Participants included midwives, nurses, physicians and health officers to ensure a diverse perspective on the mistreatment of women. The interviews were concluded after 20 interviews, as no new insights emerged, indicating data saturation.

### Data collection instruments and processes

An interview guide, aligned with the research objectives, was designed to explore the participants’ perceptions and experiences of the mistreatment of women, its drivers and its consequences. The interview guide focused on four main areas: (1) perceptions of HCPs about what women expect from healthcare facilities during maternity care, (2) experiences with different forms of mistreatment, (3) perceived factors influencing mistreatment and (4) potential impacts of mistreatment on service utilisation. Each of these focused sections of the interview guide was followed by further probing questions intended to explore the topics in more depth, based on the participants’ individual responses.

Participants were informed that their participation was voluntary. They received a participant information sheet and were asked to complete a consent form. If they chose to take part at the end of the survey responses, their response to taking part in in-depth interviews was collected separately to ensure confidentiality. All the interviews were conducted over the phone and, with permission, were digitally recorded, and all interviews took place at a mutually agreed time. HK, a PhD student at the time of data collection, conducted all the in-depth interviews in Afan Oromo. The interviewer was trained in qualitative research methods and was supervised by experienced qualitative researchers (KB, VS and AS). Following data collection, the audio recordings were transcribed and translated into English to facilitate the analysis and reporting in collaboration with English-speaking authors (KB, VS and AS). The length of the interviews ranged from 30 to 60 min.

### Data analysis

To analyse the interview findings, a hybrid technique combining inductive and deductive analyses was used [24]. In the inductive bottom-up approach, the interviews were analysed using the six stages of Braun and Clarke’s [25,26] thematic analysis to generate themes. Thematic analysis is a flexible method that helps identify key themes, richly describe large bodies of qualitative data and highlight similarities and differences in experiences [25,26]. In the deductive analysis, themes developed from the data were reassessed using the SEF and the QoC frameworks.

The interviews were transcribed verbatim into Afan Oromo and subsequently translated into English to facilitate analysis and collaboration among the English-speaking research team. To ensure the accuracy and interpretation of the large data set, the transcripts were thoroughly reviewed while listening to the corresponding audio recordings. Line-by-line coding was conducted on selected sub-samples imported into the qualitative data management software NVivo (Version 12) for coding and initial analysis. Descriptive, in-vivo and process coding methods were applied to the participants’ data, following the guidelines recommended by Saldaña [27]. Both inductive themes that emerged from the data and deductive approaches based on the interview guide were used in the analysis. Accordingly, the focus of this report is on the exploration of the drivers of the mistreatment of women during maternity care from the provider’s perspective. The study was reported in accordance with the Consolidated Criteria for Reporting Qualitative Research (COREQ) [28].

### Reflexivity

The primary author (HK) acknowledges his insider role in this context, recognising that prior experiences and interactions with HCPs in health facilities could influence both the study’s focus and the perceptions of the participants. Previous engagement in frequent discussions with HCPs about the importance of respectful maternity care and the extent of disrespectful practices has been integral. Furthermore, the primary author has previously conducted training sessions with HCPs in collaboration with the Ethiopian Midwives Association and the Ministry of Health to advance respectful maternity care. This previous engagement may have influenced the behaviours or responses of participants during the research, potentially leading them to exhibit more respectful behaviours to align with the research team’s expectations. While participants were encouraged to discuss their experiences openly, some participants may have agreed with the questions rather than sharing their true views and experiences, which might have made it harder to understand the actual mistreatment scenarios fully. To address such instances, this study consistently maintained neutrality and critically reflected on personal positionality by journaling and listening to the recordings, asking open-ended questions, avoiding leading questions and being non-judgmental throughout the interview.

While conducting this study, the researcher, as an insider, acknowledges the presence of contributing health systems and individual factors in the area. However, the primary researcher and the research team firmly believe that there should be no justification for mistreatment. It should not be tolerated and requires collaborative efforts from all local and international actors.

## Results

Overall, 20 interviews were conducted with maternal HCPs. The interviews included twelve midwives, four nurses, two health officers, and two physicians working across two hospitals and six health centres. Their clinical experience in providing maternity care ranged from 3 to 15 years.

Following thematic analysis, five sub-themes were identified as drivers of the mistreatment of women during maternity care in health facilities, and these were collectively categorised under three broader themes associated with the health system-related issues and social norms influencing individual perceptions.

### Collective categories of drivers per the SEF and QoC frameworks

**Figure 1.**
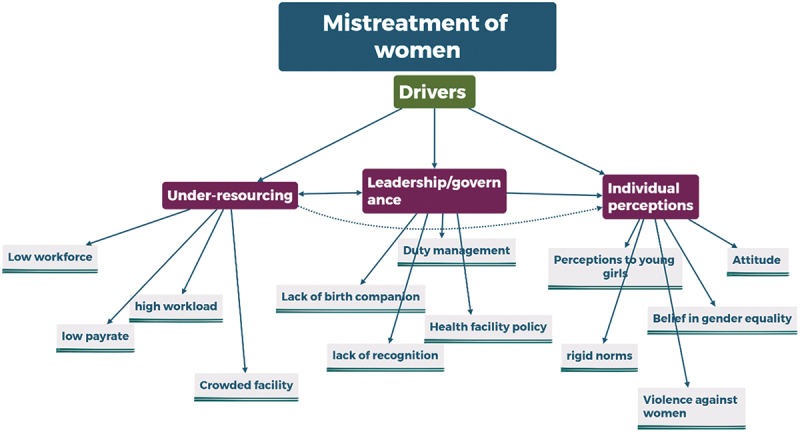
Drivers of mistreatment of women during maternity care based on the socioecological and quality of care frameworks.

The driving factors contributing to mistreatment during maternity care are classified using the SEF and QoC frameworks. The factors discussed in the following section are proximal drivers at the personal, interpersonal, health facility/system and societal levels contributing to the mistreatment of women. Although these factors are categorised under distinct themes based on thematic analysis, they often interact and may originate from broader underlying issues. These drivers are broadly classified into three categories: resource-related issues, leadership and governance and individual perceptions towards social norms, as illustrated in Figure 1.

Issues such as underpayment, a limited number of midwives or other health professionals and overcrowded facilities due to high patient flow all stem from a common root cause, under-resourcing within the healthcare system. Conversely, concerns raised by individual professionals, such as a lack of recognition and perceived unfair treatment, along with poor shift management at facility and system levels, are tied to governance challenges. Additionally, personal biases and behaviours displayed by HCPs, such as prejudices against young or unmarried women and a lack of supportive attitudes towards women overall, can be attributed to the broader issue of gender-based violence and entrenched societal norms. These themes are further synthesised and discussed in the discussion section, where they are connected to the existing body of the literature.

### Healthcare providers’ views on the nature of mistreatment and sub-themes of the drivers

**Figure 2.**
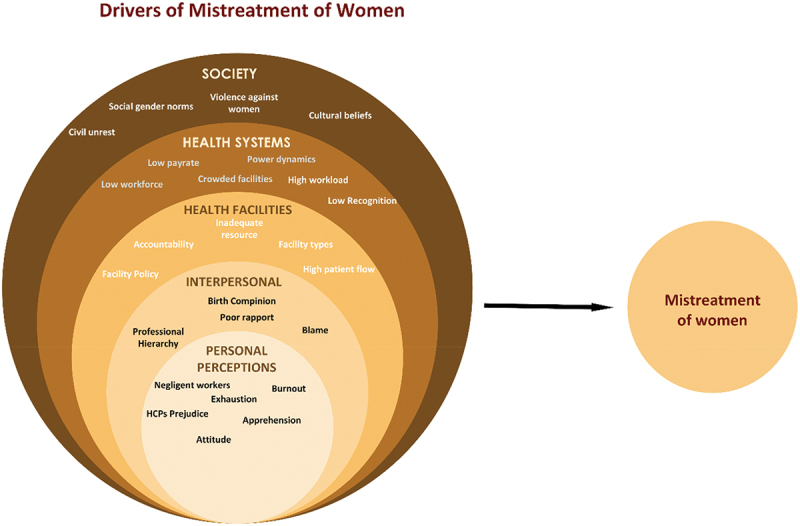
Drivers of mistreatment of women during maternity care at various levels, according to socioecological and quality of care frameworks.

Most HCPs expressed their belief that they have been providing quality care; nevertheless, concerns related to mistreatment appeared spontaneously throughout the interview discussions. Most participants reported witnessing or hearing about the various forms of mistreatment in health facilities, including physical, verbal, as well as neglect and abandonment. They described these situations as difficult for mothers seeking maternity services. One midwife explained that abuse or other forms of mistreatment often arise when women’s/family’s expectations do not align with the reality of what they experience during maternity care. Overall, drivers of mistreatment of women during maternity care were classified into the following sub-themes, ranging from personal perceptions, interpersonal, health facility and health system level issues and societal norms and conditions leading to mistreatment, as illustrated in Figure 2.

### Personal perceptions

One of the personal factors raised as contributing to the mistreatment by HCPs was the individual providers’ perceptions related to the age of the women attending care. In instances where teenage or young women arrived at the facility to give birth, some providers mentioned the presence of feelings of anger towards the situation of bringing a baby into the world at such a young age, as well as showing hostile behaviour towards young women. They reported that such reactions overwhelmed the women with the importance of understanding that having a child at such a young age was not ideal. One midwife stated that such occurrences could be aimed at advising the young women involved; however, it goes beyond normal conversation and leads to abuse:

… Sometimes, incidents like these occur. For example, comments about a mother’s age when she might be considered too young for certain responsibilities. Providers might advise her, saying things like, “Why did you get pregnant at this age? This is the time when you’re expected to focus on education and personal growth.” While advice for the future might be intended as constructive, at times, it exceeds reasonable boundaries, leading to disagreements. (IDI-HCPs-02).

Lack of cooperation from women, particularly in scenarios involving young women or when women were perceived as ‘uncooperative individuals’, was also identified as a contributing factor to physical or verbal abuse. HCPs described instances where young pregnant women with unwanted pregnancies were reluctant to have the child but still presented at the facility for birth. In some cases, midwives recounted using forceful birth assistance, which they deemed necessary to save the baby’s life. Additionally, there were reports of some women displaying indifference or explicitly expressing a desire not to give birth to a live baby. Consequently, interventions by midwives and other HCPs sometimes escalated into physical and verbal altercations:

… there are times when we seek help from another professional to safeguard the child’s well-being. For instance, in a situation where the mother intends harm, we have to call for assistance promptly and they help as in holding her legs here and there … and we assist like that because she intends to harm the child. (IDI-HCPs-01)

#### Interpersonal

Lack of cooperation and failure to comply with HCPs were cited as factors contributing to mistreatment. One participant implied that some women do not perceive or respect HCPs as professionals, which can foster mutual disrespect, leading to a normalisation of such behaviours between women and HCPs. A participant described this reciprocal tension as:

When mothers receive orders, they don’t comply to that, it can stem from various reasons maybe. Uhm, when these mothers don’t comply with the providers’ instructions, it often results in conflict or disagreement. I believe this happens because some mothers may not fully accept or respect the provider as an expert professional, which in turn affects the service provided to them. (IDI-HCPs-12)

Another interpersonal issue contributing to mistreatment was the aggressive behaviour displayed by professionals towards women and their families when conflicts arise with health facility management. Aggression is not limited to verbal or physical abuse; it can also take the form of neglect in service provision. For instance, some professionals reported instances where repeated requests from women or their families were ignored, particularly during night shifts, when staff sometimes prioritised ‘sleeping’ over fulfilling their professional responsibilities. One midwife noted that dissatisfaction with night duty pay often resulted in unacceptable behaviour, such as ignoring mothers’ requests for support and care:

… when their night shift pay is not provided as legally agreed, they express frustration towards the service … some may resist providing services and choose to sleep. But it is incorrect to attribute all the harm to mothers and children solely for personal reasons; it is seeking benefits or simply laziness which causes the problem to mothers and children. (IDI-HCPs-02)

It is well known that effective communication between HCPs and women and their families is crucial for ensuring quality care and the well-being of mothers and newborns, especially during critical periods, such as childbirth and postnatal care. Case handover between professionals during shift changes is a crucial communication method, but poor handover practices can result in the neglect of both mother and baby. One provider shared an incident where a baby became critically ill due to delayed initiation of early breastfeeding:

Yes, that’s unfortunately a real scenario we encountered. There was a situation where one midwife attended a birth around 5:30 PM but left before handing it over to the duty midwife. The newborn didn’t start feeding, and the incoming midwife was unaware of the situation. Around 8:00 PM, upon checking, the baby was in a critical condition due to the lack of feeding until that point. (IDI-HCPs-01)

### Health facility and health system

Some participants highlighted the shortage of HCPs in maternal and child health units. One midwife noted that only two midwives were available per shift to manage antenatal, birthing and postnatal care in their health centre. Consequently, this shortage often results in many pregnant women not receiving the necessary care they require and being abandoned at the antenatal care unit, resulting in dissatisfaction. As a result, women may hesitate to return to the facility after experiencing inadequate services during previous visits.

… from the follow-up stage, there were instances where, for example, we were two individuals at our station [two midwives at health centre]. One works at night, and the other works during the day. If the one who works during the day attending labour and birth, it means that mothers who come for pregnancy follow-ups end up disappointed … a risk that those in follow-up left with nobody to give care. When they come today and miss out, and then come tomorrow and waste their time … this leads to a decline in Antenatal Care (ANC) attendance as a reason. (IDI-HCPs-01)

In addition to low staffing numbers of the HCPs trying to provide care, high patient flow numbers were also reported as a challenge to respectful care:

The patient flow often exceeds expected levels. For instance, during the night shift, when there is just one midwife available, there might be five or six births. There is usually only one midwife, and when there are three women in active labour, complications like fetal distress or maternal bleeding strain the available resources. Even when additional providers are called in (health officers on call), this problem persists, especially during evening shifts, causing challenges in the provision of professional care. (IDI-HCPs-06)

Participants also raised concerns about perceived insufficient compensation among midwives who manage busy and high-risk night shifts. Midwives reported working in particularly demanding and unsupported environments compared to other professionals, whose workloads were often lighter than those in labour and birth units. They emphasised that managers failed to acknowledge their exhausting working conditions, despite receiving the same or less compensation than other HCPs. These challenging conditions are often framed as serving the community rather than being fairly compensated for their work. One midwife reflected on this issue, stressing that the health system administration lacks the ‘moral stance’ to recognise providers’ efforts adequately:

… Working all night with a high level of risk for multiple births should be appropriately compensated, but unfortunately, it’s not. When compared to someone who rests the whole night, a midwife standing through the challenges of attending five or six babies overnight, facing significant risks, receives minimal consideration for their efforts and benefits … we are serving because of the willingness to serve the community, the government lacks the moral stance to acknowledge and provide adequate payment. (IDI-HCPs-06)

A lack of sufficient attention and recognition for midwives, as well as other HCPs, was identified as a demotivating factor contributing to a reduced commitment. Limited and insufficient professional development opportunities were also highlighted, with one participant noting that offering only two educational opportunities per year creates a desperate situation, particularly when career advancement is tied to earnings:

… there is a lack of diverse opportunities for health professionals. For example, in East Wollega, with about fifty health centres and five hospitals, there are only two midwifery educational opportunities available each year. This shortage poses a serious problem. (IDI-HCPs-02)

A lack of attention stemming from governance issues was also considered the underlying cause of mistreatment. These systemic issues were seen as catalysts for disappointment, dissatisfaction with service delivery and overall provider disenchantment. This is compounded by both overcrowded and overworked conditions, significantly exacerbating mistreatment, despite providers’ intentions and efforts to meet women’s expectations. These systemic challenges created turmoil and tested both providers and patients. One provider highlighted this issue, noting that healthcare workers are neglected by the government and receive less attention compared to other public sector employees, such as teachers:

The health professionals are not compensated adequately, at least not at the level of diploma teachers, for their efforts. Providers face severe good governance issues within the district and zonal health offices. The federal government doesn’t prioritise the needs of health workers; instead, attention and resources seem to be directed towards its cabinets. With the work solely led by managers, this lack of support leaves no avenue for professionals to voice their grievances or concerns. (IDI-HCPs-19)

### Community and society

Another important theme to emerge from the analysis was community and society-related drivers. Participants highlighted how systemic and societal factors can contribute to mistreatment and impact upon the quality of care provided. One midwife recounted a tragic incident where road closures between the health centre and the hospital resulted in a delay reaching the referral centre, leading to the loss of a mother’s life. In another instance, the same midwife described a mother in prolonged labour, who had been transported to the hospital on a cart due to the lack of proper transport, resulting in the same tragic outcome. These challenges underscore the critical and indispensable roles of community infrastructure in supporting equitable healthcare delivery:

So, she experienced prolonged labour and when referred from the health centre, they had nothing to transport her with, so they took her to the Arjo Hospital in a cart. Uhm…She did not stay for more than an hour; as soon as she got off the cart and entered the hospital … she passed away. It was first birth. (IDI-HCPs-01)

## Discussion

This study explored the factors driving the mistreatment of women during maternity care in Western Ethiopia from the perspectives of HCPs. These factors stemmed from professionals’ biases towards women’s personal characteristics and societal norms rooted in gender inequality. Additionally, mistreatment was linked to interpersonal interactions, resource constraints within health facilities, the broader health system governance issues through which services are delivered and the societal context shaping maternity care in Ethiopia.

Physical and verbal abuse and discrimination against women in health facilities share characteristics with broader societal violence against women, rooted in structural gender inequality, where women are perceived as subordinate in society [13]. For example, age-related prejudice leading to the mistreatment of young women in this study could reflect societal views on marital status and sexuality. Interviews with HCPs revealed that younger women were more vulnerable to verbal and physical abuse, often due to judgements about pre-marital sexual activity and early pregnancy. Despite the legal marriage age being 18 [29], many girls marry earlier due to cultural norms, gender inequality, and societal pressures, including those in the study area and Ethiopia [20,30]. Early marriages often lack consent, and it is thought that many young women may accept marriage to avoid being labelled as ‘unwanted’ in society [31]. Although laws against child and forced marriages exist in Ethiopia, enforcement is weak and violence against women remains normalised, leading to the manifestation and acceptance of violence within health facilities. HCPs, despite being aware of community violence, often mistreated young women by blaming them for early marriage and pregnancy. Midwives and other HCPs frequently question young women’s readiness for parenthood and emphasise the importance of prioritising education and personal growth over early marriage and pregnancy. While framed as advice, this is a form of verbal abuse that has been reported in other studies as well [32,33].

Additionally, HCPs often label young or first-time mothers as non-tolerant and uncooperative, using these perceptions to justify their mistreatment towards them [34]. Such behaviours may be linked to the power imbalance between healthcare workers and women, where providers are in a position of authority over those seeking care. As a result, mistreatment can occur when that power is abused or when providers displace their anger or grievances onto the women [12]. Furthermore, such abusive behaviours of the HCPs align with the violence against women in the community. The 2016 Ethiopian Demographic and Health Survey reported that 23% of the women aged 15–49 experienced physical violence, 10% experienced sexual violence, 4% faced physical violence during pregnancy, and 34% of the married women suffered physical, sexual, or emotional violence from their partners [20]. While the community tolerates various forms of violence, it is particularly intolerant of premarital pregnancy, which is socially and religiously condemned [35,36].

As observed by Bohren (5), societal norms can influence the behaviour of HCPs, who are part of the same community. Preconceived beliefs about premarital sex and pregnancy may also contribute to the mistreatment of young women. Women often experience sexual violence, which can lead to an unintended pregnancy, creating a double burden of abuse both in the community and in healthcare settings. Similarly, mistreatment based on age and a woman’s marital status can influence how HCPs treat. Some HCPs hold the belief that young, unmarried women either do not want or deserve a baby, which can result in mistreatment during pregnancy and childbirth. While data on unmarried women giving birth is limited, unintended pregnancies are common in Ethiopia, with a prevalence of 23.5% among unmarried and formerly married women [37]. This is partly due to inadequate access to contraceptives, with only 60% of the contraceptive needs being met nationwide [20]. Some HCPs rationalise mistreatment by assuming that these women might intentionally harm their babies, using verbal or physical abuse as a way to ‘protect’ the child [38–40]. However, many fail to recognise that these pregnancies could result from rape or sexual violence, in such situations where support and counselling are needed [41]. Ethical guidelines from both the International Confederation of Midwives and the Ethiopian Midwives Association emphasise the importance of providing psychological support and upholding ethical standards to ensure that no woman or girl is harmed during pregnancy or childbirth [42,43].

Another driver of mistreatment stems from chronic resource shortages, which negatively affect the emotional well-being of the HCPs. Feelings of despair and hopelessness among midwives and other HCPs were linked to the perceived lack of recognition from the government and senior leaders regarding their challenging working conditions. In addition, difficult living conditions were worsened by high inflation and an ongoing civil war, although it is important to acknowledge that mistreatment in maternity care existed prior to the conflict. In July 2023, Ethiopia’s inflation rate was 28.8%, one of the highest in Africa, while the average monthly salary for HCPs was 7301.24 Ethiopian Birr (about 200 AUD), just above the extreme poverty line of $2.15 a day [44]. Economic pressures, along with perceived low wages, pay disparities, and lack of recognition, have intensified feelings of frustration and stress among HCPs [45]. These stressors may lead HCPs to project their grievances onto women, contributing to mistreatment and normalising such behaviour.

In Ethiopia, midwives are the primary maternal and child HCPs, with an average of three midwives per health centre working 24-h shifts [46,47]. Due to the limited number of midwives, women often reported inadequate care in smaller health centres and, therefore, were often transferred to a larger hospital, where mistreatment was more commonly reported, as reported in several earlier studies [48–50]. In addition to a national shortage of midwives, they also face significant challenges, including high workloads, lack of professional recognition, and blame for negative birth outcomes. They are frequently overlooked as essential workers in the health system. Similar findings have been found in other low-and middle-income countries [51]. Midwives experience stressors that lead to frustration, feelings of helplessness, and diminished self-worth. A power imbalance also exists within the health system, where doctors are viewed as superior to nurses and midwives, resulting in further disparities in treatment, incentives, and allowances. A report from The World Bank (2023) indicates that medical specialists earn 77% more than midwives and nurses, reinforcing the occupational and class divide. This inequality, combined with the psychological strain of negative working experiences, could be a factor contributing to the mistreatment of childbearing women in Ethiopia’s health facilities, though it should not displace the responsibility or excuse such behaviour as widely reported in the previous literature [12,51–53].

The findings suggest that while various factors may contribute to mistreatment, such behaviour cannot be justified under any circumstances. To avoid such intentional occurrences of mistreatment and justifications given for it, it is needed to sustain awareness initiatives led by professional associations and the Ministry of Health. Addressing healthcare workers’ concerns around job fairness, recognition, and the creation of supportive environments that promote mutual respect between providers and clients is indispensable.

### Strengths and limitations study

This study highlights that the factors contributing to mistreatment are complex and multifaceted, extending beyond individual provider behaviour or interpersonal misunderstandings. While the findings address key issues related to violence against women, resource limitation, and governance, a more comprehensive understanding could have been achieved by including views from community representatives and health facility managers. However, including community representatives and facility managers was not possible partly due to the civil war hindered our ability, as it was also not possible to conduct in-person interviews and focus-group discussions as originally planned. The study took place during a period of civil unrest in the area, significantly impacting the conditions for conducting the research, particularly during the data collection phase. The security situation posed serious challenges, making it difficult to move freely between districts and execute the study as initially planned, especially in engaging participants from rural areas. This contributed to the decision to perform the interviews by phone for safety reasons. This methodological adaptation raises awareness of potential limitations, such as the reduced capacity to capture non-verbal cues. This shift prompts reflection on the balance between accessibility and nuanced data collection in these challenging contexts. Nonetheless, to our knowledge, this paper is the first to explore the influence of social norms, health system failures and constraints, and midwives’ experiences on the mistreatment of women during maternity care while also emphasising the need for collaborative efforts to eliminate the mistreatment of women, their babies, as well as their families as a whole during maternity care – during antenatal care, birth, postnatal care and beyond.

Overall, trustworthiness of this study was maintained through clear documentation, peer discussions, and careful handling of transcripts to ensure accurate representation of participants’ views. The findings of this study are specific to this study setting, and it is not possible to refer it to other settings without careful consideration of the study setting described in methods section.

## Conclusions

Mistreatment of women during maternity care in health facilities was found to stem from broader issues arising from health system failures which include under-resourcing at both the health facility and system levels and leadership challenges that influence HCPs’ behaviour and power imbalances between women and staff, as well as between various cadres and social norms influencing staff behaviour. These findings highlight the need for comprehensive, multilevel interventions. Addressing gender inequality, improving HCPs motivation and ensuring equal treatment among various professionals are all crucial for promoting respectful maternity care and improving the overall quality of maternity care. Fostering a supportive health system that engages with the community, supports and values caregivers, develops health policies and strives to eliminate gender-based violence is indispensable for improved service delivery and promoting respectful maternity care.
